# Multivariate Analysis of Key Taste Compounds in Soy Sauce and Model Construction for Its Saltiness Intensity

**DOI:** 10.3390/foods14244182

**Published:** 2025-12-05

**Authors:** Shiyu Li, Junjie Yin, Li Liang, Lili Zhang, Yuyu Zhang

**Affiliations:** 1 Key Laboratory of Geriatric Nutrition and Health, Beijing Technology and Business University, Ministry of Education, Beijing 100048, China; 2 Food Laboratory of Zhongyuan, Beijing Technology and Business University, Beijing 100048, China; 3 Key Laboratory of Flavor Science of China General Chamber of Commerce, Beijing Technology and Business University, Beijing 100048, China

**Keywords:** soy sauce, taste compounds, saltiness intensity, linear regression, model construction

## Abstract

Saltiness is a key characteristic that influences the quality of soy sauce. Currently, the evaluation of saltiness intensity in soy sauce relies on sensory evaluation methods, while a scientific and efficient model to quantify saltiness is lacking. Liquid chromatography–mass spectrometry (LC-MS) and inductively coupled plasma–optical emission spectrometry (ICP-OES) were used to analyze key taste compounds in 10 soy sauce samples. According to sensory evaluation and taste addition experiments, the model for assessing the saltiness intensity of soy sauce was established. The results indicate that the umami amino acid (aspartic acid, 2.44~15.30 mg/mL; glutamic acid, 8.29~67.94 mg/mL) and the nucleotides 5′-inosinic acid (0.44~12.5 mg/mL) and 5′-guanylic acid (0.41~2.51 mg/mL) mainly contributed the saltiness intensity of soy sauce through umami synergy. Pyroglutamic acid (19.6~79.22 mg/mL) and lactic acid (0.77~0.85 mg/mL) are the primary taste-contributing organic acids to balance the taste profile in soy sauce. The Na^+^ (54.6 mg/mL) and K^+^ (8.13 mg/mL) contents are both relatively high, directly affecting the saltiness of the soy sauce. Through Spearman’s correlation analysis, 11 key taste compounds were identified to construct a multiple linear regression prediction model for saltiness intensity. The model demonstrates excellent predictive performance, providing a theoretical basis and methodological support for objectively evaluating soy sauce saltiness and reducing salt content through a scientific approach.

## 1. Introduction

Soy sauce is a traditional Chinese fermented condiment with a history spanning over three thousand years, serving as an indispensable component of global culinary culture [1]. Soy sauce sales in China reach 5 million tons annually, accounting for 60% of global soy sauce consumption, with major consumer countries including South Korea, Japan, Southeast Asian nations, and parts of North America and Europe [2]. Soy sauce is typically made primarily from soybeans, supplemented with wheat or flour. Through fermentation by microorganisms, such as lactic acid bacteria and yeast, the proteins and starch in the raw materials are hydrolyzed into various taste compounds, including amino acids, nucleotides, and organic acids [3]. Following complex biochemical reactions, the final product is a liquid sample characterized by its rich taste and reddish hue [4]. Sodium chloride (NaCl) is one of the key ingredients influencing soy sauce fermentation, serving to control microbial growth, inhibit the proliferation of unwanted bacteria, regulate fermentation speed, and influence the taste profile of the soy sauce. Traditional soy sauce contains 15% to 20% salt, which enhances its preservative properties and imparts its distinctive salty taste [5]. Excessive NaCl intake will increase the burden on the kidneys and raise blood pressure. Prolonged consumption can also increase the risk of diseases such as cardiovascular disorders and chronic kidney disease. Therefore, reducing salt content in soy sauce is a key direction for ensuring the health of consumers, and it aligns with global public health strategies aiming to lower population-wide salt intake for chronic disease prevention [6]. Research indicates that the taste profile of soy sauce exhibits remarkable complexity [7]. Its overall taste profile stems from a combination of the saltiness provided by NaCl and multiple taste-enhancing components. These substances collectively form the taste foundation of soy sauce, directly determining its taste quality and consumer acceptance.

The current research on taste compounds in soy sauce both domestically and internationally primarily focuses on free amino acids, nucleotides, organic acids, and volatile components. Qualitative and quantitative analyses have been conducted using high-performance liquid chromatography (HPLC), gas chromatography–mass spectrometry (GC-MS), liquid chromatography–mass spectrometry coupling (LC-MS), and others [8]. Research indicates that taste compounds significantly influence the overall taste profile of soy sauce, particularly its saltiness intensity. Although some studies have identified specific amino acids and peptides with the potential to enhance saltiness or to replace sodium salts in low-salt systems, current approaches primarily rely on quantitative description analysis (QDA), correlation analysis, and multivariate statistical methods to analyze the relationship between substances and taste [9]. However, existing research primarily focuses on the taste contributions of individual compounds, lacking systematic screening of key saltiness factors and in-depth analysis of multi-component synergistic effects. The evaluation of saltiness intensity remains highly dependent on human sensory evaluation, exhibiting insufficient repeatability and objectivity. Reliable predictive models based on instrumental data have yet to be established [10].

To address the challenges of sensory dependence, subjectivity, and quality control in the traditional evaluation of soy sauce saltiness, this study innovatively develops a saltiness intensity prediction model based on instrumental data and sensory evaluation data. Tiago et al. used ICP-OES technology to precisely quantify the major and trace elements in food and combined multivariate statistical analysis with sensory evaluation data to verify the applicability of this technical framework for the assessment of nutritional indicators related to food flavor [11]. Wen et al. combined sensory evaluation and ultra-high-performance liquid chromatography coupled with linear ion trap–Orbitrap tandem mass spectrometry (UHPLC-LTQ-Orbitrap-MS/MS) to explore the correlation between the taste characteristics and chemical composition of representative fractions [12]. In the study, first, LC-MS and ICP-OES were employed to quantitatively analyze the key taste compounds in soy sauce samples, including amino acids, organic acids, nucleotides, and inorganic salt ions. Second, based on the established saltiness evaluation method, sensory intensity data of saltiness for 10 types of soy sauce samples were collected. Subsequently, variables were screened through Spearman correlation analysis and taste addition experiments to identify the key taste-active substances. Finally, a saltiness intensity prediction model was constructed using multiple linear regression analysis. The model’s reliability was directly verified through statistical parameter analysis methods, and its practical prediction accuracy was further verified based on the prediction results of the saltiness of unknown samples.

## 2. Materials and Methods

### 2.1. Materials

Ten soy sauce samples marketed in China in 2025 were selected for this research. They were coded as Z10-1, Z10-2, Z10-3, Z10-4, Z10-5, Z10-6, Z10-7, Z10-8, Z10-9, and Z10-10. All samples were purchased from online marketplaces [13]. Salt (99.1%) was purchased from the China National Salt Industry Group Co., Ltd. (Beijing, China). Methanol and acetonitrile (both HPLC grade) were purchased from Fisher Scientific (Shanghai, China). Ultra-pure water was purchased from Wahaha (Hangzhou, China). Pyruvic acid, tartaric acid, malic acid, fumaric acid, lactic acid, fumaric acid, succinic acid, oxalic acid, citric acid, ascorbic acid, glycine (Gly), alanine (Ala), valine (Val), leucine (Leu), isoleucine (Iso), phenylalanine (Phe), proline (Pro), tryptophan (Try), serine (Ser), tyrosine (Tyr), cysteine (Cys), methionine (Met), threonine (Thr), aspartic acid (Asp), glutamic acid (Glu), lysine (Lys), arginine (Arg), histidine (His), cystine (Cys), 5′-guanylate disodium (5′-GMP), 5′-inosinic acid disodium (5′-IMP), cytidine 5′-monophosphate (5′-CMP), 5′-uridylic acid (5′-UMP), 5′-adenylate disodium (5′-AMP) and potassium chromate (all AR grade) were purchased from Macklin Reagent Company (Shanghai, China). Ammonium formate (chromatography grade) was purchased from Anpel Experimental Technology (Shanghai, China). 3-Nitrophenylhydrazine hydrochloride, EDAC hydrochloride, pyridine (all HPLC grade), nitric acid (premium reagent) and silver nitrate (99.9%) were obtained from Sigma-Aldrich (St. Louis, MO, USA).

### 2.2. Establishment of the Soy Sauce Saltiness Evaluation Method

All sensory experiments were approved by the Ethics Committee of the Beijing Technology and Business University (BTBU202356, Beijing, China, January 2024–December 2026). Sixty sensory evaluators (20 to 30 years of age, 20 males and 40 females) without rhinitis and possessing normal olfactory function were recruited, all of whom had extensive sensory evaluation experience and were familiar with the QDA method. All panelists provided written informed consent before the experiment. Following training designed to enhance their perception of saltiness, the panelists were able to accurately distinguish and score varying concentrations of NaCl solutions at room temperature. In the experiment, samples were coded using three random numbers. Between evaluations of each sample, the panelists rinsed their mouths with purified water to prevent cross-contamination. Each round of evaluation was conducted at one-hour intervals to avoid fatigue. Each sample group was tested in triplicate, and the results were averaged. At room temperature, the panelists conducted sensory scoring on NaCl solutions ranging from 1.0 to 9.0 g/L to quantify the perceived intensity of saltiness, following which perception curves were plotted [14]. Each sample was assessed in triplicate. The final score is based on the average value after three replicates.

### 2.3. Soy Sauce Sensory Evaluation

Ten evaluators (20 to 30 years of age, 5 males and 5 females) without rhinitis and possessing normal olfactory function were selected for taste profile analysis of soy sauce using the QDA method under room-temperature conditions. Due to the excessive saltiness of undiluted soy sauce, it was diluted 50 times prior to sensory analysis [15]. The saltiness of the soy sauce was evaluated using a linear scale. The concentrations of NaCl were 1.0 g/L (1 point), 3.0 g/L (3 points), and 6.0 g/L (6 points). The ratings for other tastes (sourness, sweetness, bitterness, and umami) were on a 6-point scale (with 0~2 indicating weak, 3~4 indicating medium, and 5~6 indicating strong). Ultimately, radar charts depicting a taste profile of the soy sauce samples were plotted by analyzing the collected data.

### 2.4. Physicochemical Characteristic Analysis of Soy Sauce

A pH meter (OHAUS, Shanghai, China) was used for pH measurement. The water activity (Aw) and non-salt soluble solid (NSSS) content of the soy sauce sample were determined according to the Chinese standards of GB 5009.238—2016 [16] and GB/T 18186—2025 [17].

The color of the soy sauce (L* (luminance), a* (red/green), and b* (yellow/blue)) was determined using a spectrophotometer, which was preheated and calibrated [18]. The chroma C* metric was calculated using Formula (1):

(1) $$C^{*}=\;\sqrt{a^{*2}+b^{*2}}$$

### 2.5. Taste Component Analysis

#### 2.5.1. Free Amino Acid Analysis

The contents of 18 free amino acids were determined using LC-MS. The soy sauce samples were diluted 100-fold with water. Subsequently, 100 μL of the diluted sample was added to 300 μL of methanol and vortexed for 60 s to mix thoroughly. The mixture was centrifuged at 17,000× *g* for 15 min at 20 °C. The supernatant was filtered twice through a 0.22 µm nylon membrane filter prior to analysis. Chromatographic separation was performed using a Waters ACQUITY ultra-high-performance liquid chromatography system with a BEH Amide column (1.7 μm, 3.0 × 100 mm) at a column temperature of 40 °C. Mobile phase A consisted of a 10 mM ammonium formate and 0. 1% formic acid aqueous solution, while mobile phase B comprised a 10 mM ammonium formate and 0.1% formic acid aqueous solution in 90% acetonitrile. The flow rate was 400 μL/min. The injection volume was 1 μL. Mass spectrometry analysis was performed using an AB Sciex 4500 triple-quadrupole mass spectrometer in electrospray ionization positive mode with multiple reaction monitoring scanning. The ion source temperature was set at 500 °C, and the spray voltage was 4500 V [19].

#### 2.5.2. Nucleotide Analysis

Five nucleotides, namely 5′-CMP, 5′-GMP, 5′-UMP, 5′-IMP, and 5′-AMP, were quantified using LC-MS methodology. First, 400 μL of water and 300 μL of 75% methanol solution were sequentially added to 100 μL of sample. The mixture was vortexed for 60 s and then centrifuged at 20 °C and 17,000× *g* for 15 min. The supernatant was filtered twice through a 0.22 µm nylon membrane filter prior to analysis. Chromatographic separation was performed using a Waters ACQUITY ultra-high-performance liquid chromatography system with a BEH Amide column (1.7 μm, 3.0 × 100 mm) at a column temperature of 40 °C. Mobile phase A was an aqueous solution of 10 mM ammonium formate and 0.1% formic acid; mobile phase B was an aqueous solution of 10 mM ammonium formate and 0.1% formic acid in 90% acetonitrile. Gradient elution was performed at a flow rate of 400 μL/min with an injection volume of 1 μL. Mass spectrometry analysis was performed using an AB Sciex 4500 triple-quadrupole mass spectrometer in electrospray ionization negative mode with multiple reaction monitoring scanning. The ion source temperature was set at 500 °C, and the spray voltage was −4500 V [19].

#### 2.5.3. Organic Acid Analysis

The LC-MS method was adopted to quantitatively analyze 10 organic acids, including malic acid, ascorbic acid, oxalic acid, succinic acid, anhydrous citric acid, tartaric acid, pyroglutamic acid, lactic acid, fumaric acid and pyruvic acid. The soy sauce sample was diluted 100-fold with deionized water. Then, 300 µL of methanol was added to 100 µL of the diluted sample, and the mixture was vortexed for 60 s, followed by centrifugation at 17,000× *g* at 20 °C for 15 min. The supernatant was filtered twice through a 0.22 µm nylon membrane filter prior to analysis. Then, derivatization was performed. First, 10 μL of 200 mM 3-nitrophenylhydrazine hydrochloride solution and 10 μL of methanol solution containing 96 mM EDAC and 6% pyridine were added to 30 μL of filtrate. Then, this mixture was derivatized at 30 °C for 60 min. Chromatographic separation was performed using a Waters ACQUITY ultra-high-performance liquid chromatography system (Waters ACQUITY, Milford, MA, USA) with a C18 column (1.7 μm, 2.1 × 100 mm) maintained at 40 °C. Gradient elution was conducted using a mobile phase of 0.1% formic acid in water (A) and acetonitrile (B) at a flow rate of 400 μL/min with a 1 μL injection volume. Mass spectrometry analysis was performed using an AB Sciex 4500 triple-quadrupole mass spectrometer with multiple reaction monitoring scanning in electrospray ionization negative mode. The ion source temperature was set to 500 °C, and the spray voltage was −4500 V [19].

#### 2.5.4. Inorganic Salt Ion Analysis

Metal ions in the soy sauce were determined using ICP-OES (SHIMADZU, Kyoto, Japan). First, 0.5 mL of soy sauce sample was placed in a microwave digestion inner vessel, and 10 mL of nitric acid was added. Then, the mixture was placed in the TK12 acid digestion system, pre-digested at 100 °C for 30 min, cooled to room temperature, and subsequently subjected to microwave digestion (150 °C for 10 min and then 180 °C for 15 min). The digestion solution was evaporated to approximately 1 mL at 160 °C, transferred to a 25 mL volumetric flask, diluted to the mark with water, and mixed thoroughly to obtain the sample solution for analysis. Analysis was conducted using the ICPE-9800. Quantitative analysis of metal ion elements in the samples was performed using the standard curve method with multi-element standard solutions. All samples were analyzed in triplicate, with blank controls included [20].

The spectrophotometric method was employed to determine the chloride content in soy sauce. By reacting mercuric thiocyanate with chloride ions to form a colored complex, the absorbance was measured at a 436 nm wavelength, enabling the quantification of chloride in soy sauce samples. The procedure involved sample pretreatment, reaction incubation, and absorbance measurement, with the final chloride ion concentration calculated using Formula (2) [21].

(2) $$C_{{Cl}^{-}}=\frac{A_{\mathrm{measurement}}-A_{\mathrm{Blank}}}{A_{\mathrm{Standard}}-A_{\mathrm{Blank}}}\times C_{\mathrm{Standard}}\times N$$

In the formula, *C*_Cl^−^_ denotes the chloride ion content, mmol/L, *C*_standard_ represents the standard solution concentration,100 mmol/L and *N* indicates the dilution factor prior to sample testing.

### 2.6. Development and Validation of a Sensory Predictive Model for Saltiness Intensity

For data subjected to standardized preprocessing, Spearman’s correlation analysis and taste-blending experiments were employed to identify indicators significantly correlated with saltiness. A multiple linear regression (MLR) model was established to quantify the relationship between independent variables (*X,* including amino acids, nucleotides, organic acids, metal ions, and chloride ions) and the perception of intensity saltiness (*Y*).

The *R*^2^ (coefficient of determination), *p* values, and RMSE (root mean square error) were used as evaluation metrics to assess model performance [22]. The *R*^2^ values range from 0 to 1, with values closer to 1 indicating a better model fit [23].

### 2.7. Statistical Analysis

Using SPSS Statistics 26.0 software, we conducted one-way ANOVA for significance analysis and multiple linear regression analysis to construct the model. Duncan’s multiple range test was subsequently conducted at a significance level of 5% (*p* < 0.05).

## 3. Results and Discussion

### 3.1. Establishment of a Method for Evaluating the Saltiness of Soy Sauce

**Figure 1:**
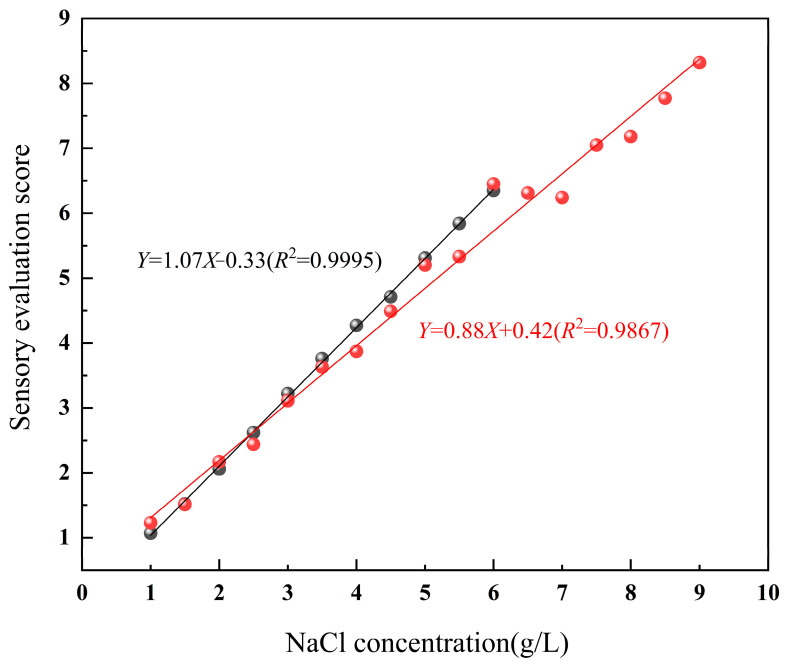
Fitted curves of saltiness perception at different NaCl concentrations (black line: 1~6 g/L; red line: 1~9 g/L).

The fitted curves of the salty taste perception intensity at different NaCl concentrations are shown in Figure 1. Within the range of 1.0~9.0 g/L, the salty taste perception intensity of NaCl exhibits a linear relationship with its concentration (red line, *R*^2^ = 0.9867). However, in actual salty taste perception, when the NaCl concentration exceeds 6.0 g/L, it approaches or reaches the level of sensory discomfort for a person. At this point, the panelists had limited ability to distinguish differences in NaCl concentrations at the 0.1 g/L level.

To verify the discriminability of saltiness perception, subsequent experiments systematically investigated the discriminative ability across high-, medium-, and low-concentration ranges using the gradient ranking method combined with QDA. Ranking experiments were conducted with different concentration gradients (0.1~0.5 g/L), and the reliable range of saltiness perception was comprehensively evaluated by integrating the assessment of sensory discomfort threshold and QDA scoring. The verification experiments showed that in the high-concentration range above 6.0 g/L, the ranking accuracy decreased significantly. Even when a gradient of 0.5 g/L was used, the accuracy was only 20%. In contrast, within the 4.0~6.0 g/L range, the ranking accuracy remained at 70% with a 0.5 g/L gradient. Based on the above results, the standard upper limit of saltiness perception was finally determined to be 6.0 g/L so as to ensure the reliability of the evaluation results.

Based on the comprehensive evaluation of salty taste perception and statistical analysis results, within the range of 1.0~6.0 g/L, the perception of saltiness exhibits a highly linear relationship with NaCl concentration (black line, *R*^2^ = 0.9995). The new salty taste evaluation criteria presented are based on NaCl concentrations of 1.0 g/L (1 point), 3.0 g/L (3 points), and 6.0 g/L (6 points), which were used to assess the saltiness of soy sauce samples. Compared with previous studies, the sensory evaluation team in this research was significantly larger. Relying on this team, we successfully established an assessment method for the saltiness intensity of soy sauce. The advantage of a large team is that it can effectively reduce individual subjective biases and improve the objectivity and stability of the results, providing scientific support for the accurate quantification of the saltiness intensity of soy sauce. This design not only ensures the high reliability of the subsequent modeling in obtaining sensory evaluations, but also represents a prominent improvement over existing studies for this method.

### 3.2. Sensory Evaluation Results for Soy Sauce

**Figure 2:**
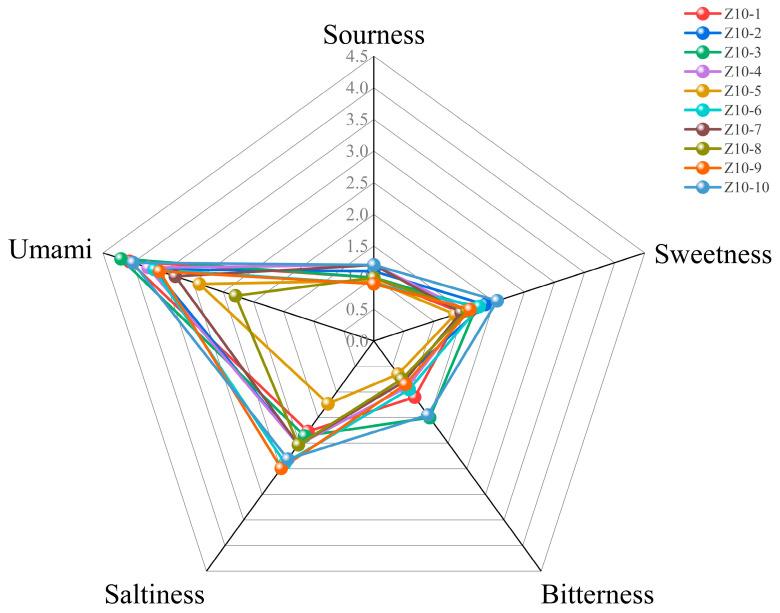
Taste profiles of soy sauce samples.

Based on the established scoring criteria, 10 soy sauce samples were diluted to an appropriate concentration range (50-fold dilution) and evaluated for taste characteristics using the QDA method, with the results shown in Figure 2. As shown in Figure 2, soy sauce, as a complex flavor condiment, is primarily characterized by salty and umami notes, complemented by sour, bitter, and sweet tastes. Typically, umami and salty tastes exhibit synergistic effects. In the present work, different types of soy sauce varied in the intensity of their taste characteristics, including saltiness, umami, bitterness, sourness, and sweetness; however, the overall differences in taste profiles primarily center on the interplay between umami and salty tastes [14]. In the saltiness dimension, Z10-9 received the highest score of 2.49 points, followed by Z10-6 (2.39 points) and Z10-10 (2.31 points), indicating that salty taste was more pronounced [5]. In terms of umami, the soy sauces with relatively high sensory scores included Z10-3, Z10-1, and Z10-4, which scored 4.20 points, 4.06 points, and 3.75 points, respectively. Notably, soy sauces Z10-3 and Z10-10 also exhibited a distinctly perceptible bitter taste profile, featuring relatively complex taste contours and greater richness. In contrast, the primary taste profiles of the three soy sauces (Z10-1, Z10-6, and Z10-9) center on saltiness and umami, forming their core taste attributes.

### 3.3. Physicochemical Characteristics

The quality of soy sauce is influenced by various physicochemical properties, including pH, Aw, and NSSS. These parameters not only affect the taste and mouthfeel of soy sauce but also impact its stability and shelf life. The results of the physicochemical analysis are shown in Table 1. The pH levels of the 10 soy sauces exhibited good consistency, remaining stable between 4.9 and 5.5. The Aw fluctuated within a narrow range (0.83~0.86), which helps inhibit microbial growth and extend shelf life. The NSSS content of the 10 soy sauce samples ranged from 0.65 to 32.49 g/100 mL, exhibiting significant variation (*p* < 0.05). This disparity may relate to peptide and amino acid composition, reflecting diverse brewing techniques. García et al. reported that the NSSS content positively correlates with soy sauce richness and may indirectly influence salt perception by affecting taste release [24].

The color of soy sauce is closely related to its taste. Part of the color in soy sauce is formed during the fermentation process, and pasteurization further enhance its color [12]. Regarding color characteristics (Table 1), the soy sauce generally exhibited low L* values (1.34~8.61). Among them, the Z10-5 had a relatively high lightness of 8.61, and its a* and b* values showed a positive correlation, indicating a darker hue. This may be related to the degree of browning during soy sauce production. The primary reasons include the Maillard reaction and enzymatic browning between reducing sugars, amino acids, peptides, and proteins during fermentation, as well as the gradual dissolution of brown substances formed during soybean steaming, leading to increased color intensity [13]. Although the color of soy sauce does not directly determine its saltiness, it may influence the perception of saltiness through visual cues.

### 3.4. Quantitative Analysis of Taste Components

#### 3.4.1. Analysis of Free Amino Acid Contents

**Figure 3:**
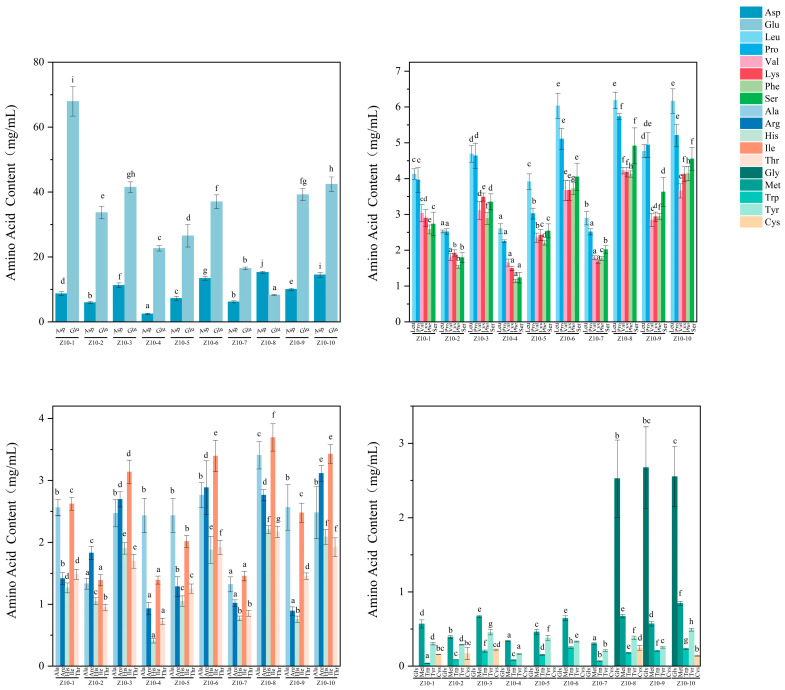
Amino acid analysis results for soy sauce samples. Different letters represent significant differences between groups (*p* < 0.05).

Free amino acids are closely linked to the taste profile of soy sauce, not only forming its taste foundation but also potentially influencing salt perception through taste interactions. They are considered key contributors to the taste of fermented condiments [25]. Research has found that the taste of soy sauce is primarily due to its peptide content and the ratio of free sweet amino acids to umami amino acids [26]. Figure 3 shows the amino acid analysis results for 10 soy sauce varieties. Each soy sauce contained 16 to 18 taste-contributing amino acids, with total amino acid content ranging from 41.31 to 106.38 mg/mL, reflecting significant differences between varieties. Among these, samples Z10-2, Z10-4, and Z10-7 exhibited relatively low total amino acid content, consistent with the grade-three soy sauce quality and lower levels of amino acid nitrogen. The total content of umami amino acids (Asp and Glu) in soy sauce (22.66~76.63 mg/mL) is significantly higher than that of sweet amino acids (8.13~22.97 mg/mL) and bitter amino acids (10.20~24.24 mg/mL). Research indicates that higher total amino acid content and umami amino acid content correlate with superior taste quality and more pronounced umami notes in soy sauce, while sweet amino acids and bitter amino acids contribute to a richer, more mellow mouthfeel [27]. The differences in Asp content among the 10 types of soy sauce were significant (*p* < 0.05). However, there was a significant difference in the proportion of Glu to total amino acids, ranging from 17% to 61%. The lowest level of glutamic acid in Z10-8 may have been due to the absence of MSG in Z10-8. Dong et al. found that the carboxyl group of Asp among umami amino acids contributes to umami characteristics, typically synergizing with Glu to form the umami taste of soy sauce [28]. The total umami amino acids in all 10 soy sauces were at relatively high levels, particularly Glu. These primarily originated from protein decomposition, with a portion also derived from the umami enhancer MSG, which was added during preparation. This likely represents the core factor behind the umami differences among the 10 soy sauces, highlighting the decisive influence of variations in production ingredients (ratios of soybeans, wheat, and rice), fermentation strains, fermentation cycles, and process parameters on the final taste profile of soy sauce. For samples Z10-6, Z10-9, and Z10-10, higher Glu content in umami amino acids correlates with higher salty taste values. This finding is consistent with the results of Zhou et al., who demonstrated a significant positive correlation between the umami intensity of soy sauce and its glutamic acid content, as well as with the findings of Lioe et al. regarding the umami fraction of Indonesian soy sauce [29,30].

Among the 10 soy sauce samples, the proportion of total sweet amino acids was relatively high, ranging from approximately 12.8% to 32.2%. Samples Z10-8, Z10-9, and Z10-10, in particular, not only effectively neutralized the sharpness of the soy sauce’s saltiness, but their higher sweet amino acid content also softened the taste, resulting in milder saltiness and improved palatability. Lys contributes to the formation of high-quality complex tastes in soy sauce [25]. Zhou et al. reported that Ala makes a significant contribution to the sweetness of both Chinese and Japanese soy sauce; Gly buffers the sharpness of saltiness, improving taste balance to enhance the overall flavor [29]. Free amino acids such as Ser and Thr, which are responsible for sweet taste, may affect the sweetness attribute through synergistic interaction with other chemical compounds in soy sauce [30]. Additionally, Pro contributes to sweetness [31]. The content of bitter amino acids in soy sauce is second only to sweet amino acids, with a total range of 10.20~24.24 mg/mL. Tyr and Trp may impart a slight bitter taste, which could compete with saltiness and diminish its perception. Val is a bitter amino acid, and the increases in bitterness help enhance the rich taste of soy sauce, making its aftertaste linger longer in the mouth [25]. Human psychophysical studies have shown that at low concentrations, Arg elicits a sensation that is mainly bitter [32]. Strongly hydrophobic amino acids, such as His, Met, Val, Leu, Ile, Phe, Trp, and Tyr, exhibit bitter tastes [33]. Among these, Leu, Ile, and Phe significantly influence the bitterness of soy sauce [29]. Additionally, Phe and Tyr possess distinctive aromas that contribute unique taste characteristics to soy sauce. The taste and rich aroma of soy sauce are closely linked to its taste-enhancing amino acids. Among these, umami amino acids dominate the taste profile, while Asp and Glu primarily influence the saltiness through synergistic effects. Sweet and bitter amino acids play supporting roles, contributing to the soy sauce’s complex and mellow taste.

#### 3.4.2. Nucleotide Content Analysis

**Figure 4:**
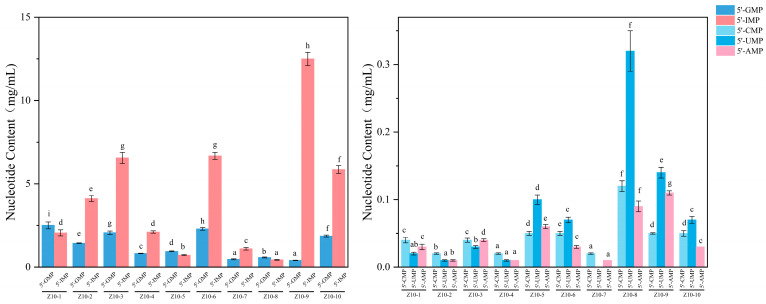
Nucleotide analysis results of soy sauce samples. Different letters represent significant differences between groups (*p* < 0.05).

5′-IMP and 5′-GMP are the primary umami components, which can be produced through microbial fermentation or added later. Adding a certain proportion to soy sauce can significantly enhance the perception of its saltiness through synergistic effects and improve the overall taste quality [34]. The nucleotide analysis results for 10 soy sauces are shown in Figure 4, with total nucleotide content ranging from 1.55 to 13.21 mg/mL. 5′-IMP and 5′-GMP are the two predominant nucleotides in the soy sauces. This finding is highly consistent with the report by Zhou et al. on the nucleotides responsible for the umami taste in soy sauce, confirming the crucial auxiliary role of nucleotides in enhancing perceived saltiness [29]. Results indicate that the saltiness of soy sauce samples positively correlates with the total nucleotide content. The high-saltiness-intensity sample Z10-9 exhibited the highest total nucleotide content (13.21 mg/mL), while the low-saltiness-intensity sample Z10-8 showed the lowest total nucleotide content (1.55 mg/mL). This difference in nucleotide content was primarily attributed to the distinct formulations of the two samples. Z10-9 was formulated with additional disodium inosinate alongside yeast extract. However, the saltiness intensity score for Z10-8 was lower than that for Z10-9. This is because the actual amount of NaCl added to Z10-8 was lower. Furthermore, the addition of monosodium glutamate in Z10-9 exhibited significant synergistic taste enhancement with disodium inosinate, substantially elevating the product’s overall taste profile. This allowed the panelists to experience intense sensory satisfaction even under low-salt conditions, amplifying the perception of saltiness and thus achieving the effect of “reducing salt without sacrificing saltiness”. This aligns with the findings of Zhang et al., who demonstrated that yeast extract, monosodium glutamate, and disodium inosinate can collectively enhance taste to partially replace salt in foods [35].

The content of 5′-IMP is particularly strongly associated with saltiness perception. As an umami enhancer, it likely indirectly amplifies the perception of saltiness through taste synergistic effects. The content of 5′-IMP in the samples ranged from 0.44 to 12.50 mg/mL. Among them, the 5′-IMP content in Z10-9 reached as high as 12.50 mg/mL, which is significantly higher than that in other samples. This aligns with its pronounced effect on the nucleotides responsible for the umami taste in soy sauce, confirming the crucial auxiliary role of nucleotides in enhancing perceived saltiness and salty taste profile in sensory evaluation, which may be attributed to additives in the soy sauce formulation. In terms of 5′-GMP content, Z10-1 (2.51 mg/mL) was significantly higher than Z10-9 (0.41 mg/mL), likely due to differences in the taste enhancers used. The pronounced umami profile exhibited by Z10-9 may be attributed to its exceptionally high 5′-IMP content. This aligns with the findings reported by Lioe et al., who observed taste interactions between 5′-IMP or 5′-GMP and Glu or Asp, both of which impart umami tastes [36]. Furthermore, this conclusion is consistent with the observation that nucleotides interact with NaCl to enhance the salty taste of soy sauce. 5′-AMP exhibits a bitter taste with very weak umami intensity. Its content in the 10 soy sauces tested remained at relatively low levels, effectively preventing its bitterness from negatively impacting the overall taste profile. This finding strongly supports the research by Bi et al., which concluded that although soy sauce bitterness is commonly perceived as an undesirable taste, it plays a crucial role in balancing the overall taste [37].

#### 3.4.3. Analysis of Organic Acid Content

**Figure 5:**
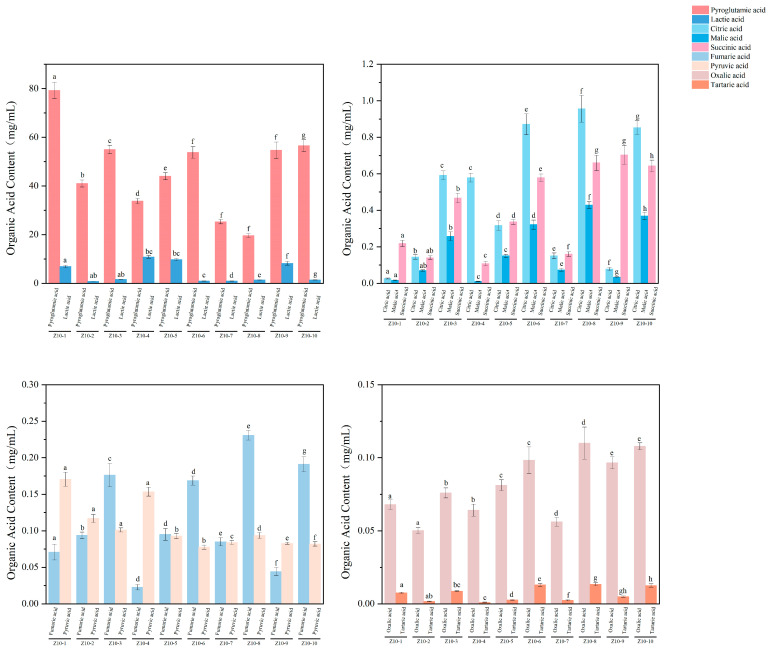
Organic acid analysis results for soy sauce samples. Different letters represent significant differences between groups (*p* < 0.05).

In soy sauce fermentation, organic acids produced by microbial metabolism serve as key taste compounds. They not only provide the fundamental sourness but also synergize with other taste components to collectively construct the unique and complex taste profile of soy sauce [27]. The organic acid analysis results for 10 soy sauce samples are shown in Figure 5. Samples Z10-1, Z10-6, and Z10-9 exhibited higher total organic acid content, likely due to extended fermentation periods that provided sufficient time for enzymatic hydrolysis of raw materials. This finding aligns with the results reported by Li et al. [38]. Among the 10 soy sauce samples, pyroglutamic acid and lactic acid were the predominant organic acids, with concentrations ranging from 19.60 to 79.22 mg/mL and 0.77 to 10.85 mg/mL, respectively, showing significant differences (*p* < 0.05). This aligns with the findings of Kong et al., indicating that organic acids influence the sourness intensity of soy sauce [39]. Among these, pyroglutamic acid and lactic acid can regulate the taste profile of soy sauce, ensuring a balanced flavor. Soy sauce contains abundant lactic acid (0.77~10.85 mg/mL), consistent with the findings reported by Lioe et al. showing that lactic acid constitutes a significant portion of soy sauce’s organic acids, accounting for 70~80% of its total organic acid content [40]. Syafaa et al. noted that sugars produced during koji fermentation in soy sauce undergo microbial metabolism to form lactic acid and acetic acid, causing the pH of the fermenting mixture to drop below 5.5 [41]. This aligns with the data presented in the present work where both sample Z10-4, with the highest lactic acid content (10.85 mg/mL), and sample Z10-5, which had an additional lactic acid addition raising its content to 9.74 mg/mL, exhibited the lowest pH values (4.81). Despite their lower pH, their sourness intensity was not particularly pronounced, likely due to the mild sourness characteristic of lactic acid. When lactic acid concentration was excessively high (Z10-4, 10.85 mg/mL), the intense sourness directly masked the salty taste, resulting in reduced perceived saltiness. Z10-1 exhibited the highest levels of both pyroglutamic acid and Glu, at 79.22 mg/mL and 67.94 mg/mL, respectively. According to Cheng et al., approximately 46% of the glutamic acid in soybean and wheat extracts exists in the form of glutamine [42]. During fermentation, this portion of glutamine undergoes irreversible conversion into tasteless pyroglutamic acid through non-enzymatic pyrolysis reactions in the absence of glutaminase activity, thereby diminishing the freshness of soy sauce.

Among the 10 types of soy sauce, the sour taste stemmed from the presence of higher concentrations of organic acids, particularly citric acid (0.03~0.96 mg/mL), which may have contributed to the overall sourness intensity of the soy sauce. Pyruvate content in the samples (0.08~0.17 mg/mL) was low, with no significant variation. As a characteristic compound of modern soy sauce, pyruvate forms during the acid hydrolysis of soybeans and serves as the starting point for the tricarboxylic acid cycle and a raw material for free amino acids [39]. Succinic acid content in the samples ranged from 0.11 to 0.70 mg/mL. It primarily contributes to umami taste and, as an intermediate in the tricarboxylic acid cycle, indirectly accelerates amino acid production. It also enhances binding with glutamic acid, further intensifying the umami taste of soy sauce [28]. Oxalic acid and tartaric acid content were both relatively low, at 0.05~0.11 mg/mL and 0.001~0.013 mg/mL, respectively, and may exert a certain inhibitory effect on the umami taste of soy sauce [28]. However, when present in appropriate amounts, the sourness intensity can enhance the overall taste balance of the product. Ascorbic acid was detected only in the Z10-8 sample (0.08 mg/mL), reflecting its distribution specificity. This may be attributed to the use of specific raw materials rich in this substance or the adoption of antioxidant protection processes, providing clues for developing functional soy sauce products [38]. Feng et al. indicate that sorbic acid, commonly used as a preservative additive in certain soy sauces, inevitably enhances the sourness of these products [43]. Despite the relatively low concentrations, these organic acids remain one of the most important taste-active substances in soy sauce. They influence the sauce’s acidity and maintain its taste balance, further indirectly affecting the perception of saltiness through taste equilibrium [39]. This enhances the sauce’s richness, making its salty sensation more enduring and mellow.

#### 3.4.4. Analysis of Inorganic Salt Ion Content

**Figure 6:**
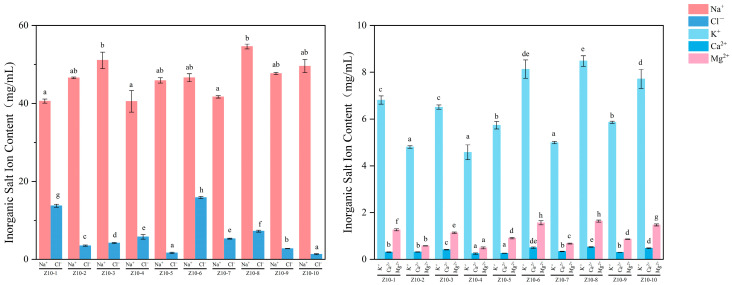
Inorganic salt ion analysis results for soy sauce samples. Different letters represent significant differences between groups (*p* < 0.05).

The content of inorganic salt ions is a characteristic that distinguishes production processes and raw material formulations. Its composition and concentration not only determine the saltiness of soy sauce but also serve as crucial indicators for comprehensively evaluating its taste profile and quality grade. The results of the systematic determination of five inorganic salt ions, namely, Ca^2+^, Mg^2+^, K^+^, Na^+^, and Cl^−^, in 10 soy sauce samples are shown in Figure 6. The Na^+^ produced by the dissociation of MSG is fully included in the total Na^+^ content, becoming one of the components contributing to the salty taste of soy sauce. Overall, Na^+^ content was most prominent (40.53~54.60 mg/mL), serving as the key ion for activating oral salt taste receptors. Its concentration variations directly governed differences in saltiness, exhibiting a strong correlation with the perception of saltiness. Consistent with the data reported by Kong et al., Na^+^ makes a significant contribution to the taste of soy sauce [39]. Moreover, the saltiness intensity score of the high-Na^+^ concentration sample Z10-8 (54.60 mg/mL) was significantly higher than that of the low-Na^+^ concentration samples Z10-1 (40.60 mg/mL) and Z10-4 (40.53 mg/mL). Results indicate that Na^+^, K^+^ (4.58~8.48 mg/mL), and Cl^−^ (1.33~15.85 mg/mL) are the three primary inorganic salt ions in soy sauce, with Na^+^ accounting for 64.00~84.25% of the total ion content. This aligns with previous studies, where Na^+^ and K^+^ served as the primary sources of saltiness. Cl^−^ itself is tasteless but acts as a direct contributor to saltiness, playing a crucial role in shaping the pure salty taste sensation. Only when an appropriate amount of Na^+^ is present will Na^+^ and Cl^−^ exhibit synergistic effects to produce a salty taste [44]. Taste reduction experiments revealed that the absence of Na^+^ significantly diminished both salty and umami tastes, indicating a synergistic enhancement effect when present alongside other taste substances [45]. The concentrations of K^+^, Mg^2+^, and Ca^2+^ also influence salt perception. These three cations can produce a faint salty taste or alter mouthfeel, but due to the concentrations being far lower than Na^+^, the direct contribution to overall saltiness is limited. They primarily affect the texture and richness of soy sauce. Significant differences in ion content were observed among different samples (*p* < 0.05). Sample Z10-8 exhibited higher concentrations of K^+^, Na^+^, Mg^2+^, and Ca^2+^ compared to other samples. Furthermore, Cl^−^ content fluctuated considerably across samples, ranging from 15.85 mg/mL in sample Z10-6 to just 1.33 mg/mL in the Z10-10 sample. This variation likely stems from differences in production processes, formulation ingredients, and salt sources among various soy sauces. It also indicates that inorganic salt ions serve as important reference indicators for distinguishing the taste characteristics of soy sauce [29].

### 3.5. Development and Validation of a Sensory Model for Saltiness Intensity

The taste profile of soy sauce results from the combined effects of multiple taste compounds. Variations in the types and concentrations of these compounds directly influence the taste quality. The taste characteristics of soy sauce are primarily shaped by compounds such as umami amino acids, bitter amino acids, nucleotides, organic acids, and sodium chloride, each contributing to the overall taste intensity to varying degrees. To investigate the relationship between saltiness intensity and the corresponding taste compounds, the taste substances of different soy sauces were measured, and using the saltiness intensity results, a correlation model was established to quantitatively evaluate soy sauce saltiness [46].

A multivariate linear regression equation was employed to construct a sensory model for saltiness intensity based on sensory evaluation data and taste components of soy sauce (amino acids, nucleotides, organic acids, and inorganic salt ions). Based on the analysis of 10 soy sauces, supplemented by taste addition experiment data from 2 additional soy sauces, a quantitative prediction model for saltiness intensity was constructed. Expanding the soy sauce sample size is necessary for enhancing the accuracy of the saltiness prediction model in future research. Through Spearman’s correlation analysis and taste-enhancement experiments, 11 compounds were identified as correlated with the saltiness intensity value *Y*. These compounds include citric acid, tartaric acid, lactic acid, succinic acid, Asp, Glu, 5′-GMP, K^+^, Na^+^, Ca^2+^, and Mg^2+^. A regression model was constructed between the saltiness intensity of soy sauce and 11 taste compounds using multiple linear regression analysis [47], as detailed in Equation (3):

(3) $$Y\;=\;2.181-0.120X_{1}+0.450X_{2}-0.132X_{3}-0.445X_{4}+0.181X_{5}+0.088X_{6}-0.134X_{7}\;+0.148X_{8}+0.035X_{9}-0.569X_{10}+0.301X_{11}$$

where *X*_1_ is the citric acid standard value; *X*_2_ is the tartaric acid standard value; *X*_3_ is the lactic acid standard value; *X*_4_ is the succinic acid standard value; *X*_5_ is the Asp standard value; *X*_6_ is the Glu standard value; *X*_7_ is the 5′-GMP standard value; *X*_8_ is the K^+^ standard value; *X_9_* is the Na^+^ standard value; *X*_10_ is the Ca^2+^ standard value; and *X*_11_ is the Mg^2+^ standard value.

To clarify the specific contribution of each variable to the overall calculation, this study employed standardized coefficients for illustration. The contributions of different variables were classified into three categories: highly influential variables (absolute value of coefficient > 0.3), moderately influential variables (absolute value of coefficient 0.1~0.3), and low-influence variables (absolute value < 0.1). X_10_ (coefficient: −0.569) acts as the strongest negative impact factor. For each 1-unit increase in X_10_, Y decreases by 0.569 units. As the most powerful predictive variable in the model, its effect is negative. X_5_ (+0.181) and X_8_ (+0.148) both had medium positive contributions. X_7_ (−0.134), X_3_ (−0.132), and X_1_ (−0.120) all exerted medium negative effects. X_6_ (+0.088) and X_9_ (+0.035) had weak positive effects.

Whether the regression model can be used for practical predictions depends on the validation of the regression model [48]. Statistical parameter analysis of the constructed salty taste sensory model revealed that the regression equation’s significance test (*p* = 0.000 < 0.01) demonstrated the model’s highly significant value. The model’s coefficient of determination *R*^2^ approaches 1, demonstrating that the regression model exhibits high precision and excellent predictive performance. The model’s coefficient of determination *R*^2^ = 0.833 indicates that the model explains 83.3% of the variation in saltiness intensity, demonstrating good fit. The root mean square error (RMSE = 0.197) is relatively small, indicating that the deviation between the model’s predicted values and the actual measured values falls within an acceptable range, meaning that the predicted values are closer to the actual values. In previous studies, Zhang et al. reported an *R*^2^ value of 0.78 for predicting the saltiness of sauces [49], while Wang et al. achieved an *R*^2^ of 0.81 for the soy sauce saltiness model based on electronic tongues [50]. The model constructed in this study demonstrates a certain advantage in terms of goodness of fit, exhibiting good precision and stability.

To further enhance the accuracy of the sensory prediction model for saltiness intensity, two unknown soy sauce samples were utilized. By comparing the sensory evaluation scores of the samples with the predicted saltiness intensity, the accuracy of the sensory model for saltiness intensity was validated. The model validation results for soy sauce samples indicate that the relative errors between predicted values and actual saltiness intensity values were both less than 10% (9% and 7%), demonstrating that the model possesses excellent extrapolation capabilities and exhibits high consistency with actual sensory evaluation outcomes. Referring to the standard established by Wang et al. in taste models for condiments, where error is typically controlled within 15%, this model demonstrates excellent predictive accuracy [46]. Based on the above validation, the salty taste sensory prediction model is deemed effective and reliable for predicting the saltiness intensity of soy sauce.

## 4. Conclusions

This study systematically demonstrates that the taste profile of soy sauce is determined by the synergistic interaction of its amino acids, organic acids, nucleotides, and inorganic salt ions. Specifically, glutamic acid and aspartic acid are the dominant amino acids responsible for umami taste, and they synergistically enhance the perception of saltiness when combined with high concentrations of 5′-IMP and 5′-GMP. Among organic acids, pyroglutamic acid and lactic acid serve as primary components contributing to the sour taste, while subtle variations in the content of organic acids, such as citric acid and tartaric acid, contribute to the overall taste balance of soy sauce. High concentrations of Na^+^ and K^+^ act as taste enhancers, not only directly imparting saltiness but also synergizing with umami compounds to influence the perceived intensity of saltiness. This study innovatively employed Spearman’s correlation analysis and taste addition experiments to screen 11 key taste compounds. Multiple linear regression analysis was used to construct a model for predicting salty taste intensity. The constructed model was validated to effectively predict the salty taste of unknown soy sauce samples. The results obtained provide a reliable method for the quantitative evaluation of soy sauce saltiness. The established saltiness intensity prediction model can be directly applied to the scientific salt reduction of soy sauce and the development of new low-sodium products. It can also be extended to the flavor evaluation and salt reduction research of other fermented condiments, providing methodological references for the health-oriented upgrading of the industry. However, the main content of the present work primarily focuses on the influence of non-volatile compounds in soy sauce on its saltiness intensity. Future work may concentrate on further explaining the influence of volatile components of soy sauce on its saltiness intensity.

## Figures and Tables

**Table 1:** Results of physicochemical properties analysis in soy sauce samples.

Physicochemical Properties	Z10-1	Z10-2	Z10-3	Z10-4	Z10-5	Z10-6	Z10-7	Z10-8	Z10-9	Z10-10
pH	5.40 ± 0.00 ^f^	5.16 ± 0.01 ^d^	5.33 ± 0.03 ^e^	4.81 ± 0.01 ^a^	4.81 ± 0.01 ^a^	5.39 ± 0.00 ^f^	4.99 ± 0.01 ^b^	5.05 ± 0.01 ^c^	5.40 ± 0.01 ^f^	5.37 ± 0.03 ^f^
Aw	0.85 ± 0.00 ^g^	0.86 ± 0.00 ^i^	0.83 ± 0.00 ^a^	0.85 ± 0.00 ^f^	0.84 ± 0.00 ^d^	0.83 ± 0.00 ^c^	0.86 ± 0.00 ^h^	0.83 ± 0.00 ^ab^	0.85 ± 0.00 ^e^	0.83 ± 0.00 ^b^
NSSS (g/100 mL)	16.32 ± 0.87 ^d^	1.40 ± 1.15 ^a^	13.59 ± 2.62 ^cd^	0.65 ± 0.21 ^a^	32.49 ± 0.22 ^f^	14.12 ± 0.31 ^d^	7.53 ± 0.66 ^b^	20.91 ± 0.11 ^e^	10.78 ± 0.77 ^c^	14.43 ± 0.28 ^d^
L*	3.25 ± 0.01 ^f^	4.01 ± 0.00 ^h^	2.51 ± 0.01 ^c^	4.28 ± 0.02 ^i^	8.61 ± 0.01 ^j^	2.05 ± 0.01 ^b^	3.89 ± 0.01 ^g^	2.99 ± 0.01 ^d^	3.23 ± 0.00 ^e^	1.34 ± 0.00 ^a^
a*	18.67 ± 0.03 ^e^	22.52 ± 0.01 ^i^	15.19 ± 0.01 ^c^	21.91 ± 0.01 ^h^	31.05 ± 0.02 ^j^	11.76 ± 0.01 ^b^	21.04 ± 0.01 ^g^	17.90 ± 0.01 ^d^	18.72 ± 0.01 ^f^	7.98 ± 0.01 ^a^
b*	5.41 ± 0.01 ^f^	6.85 ± 0.01 ^h^	4.27 ± 0.00 ^c^	7.01 ± 0.01 ^i^	14.34 ± 0.03 ^j^	3.26 ± 0.01 ^b^	6.47 ± 0.01 ^g^	5.07 ± 0.01 ^d^	5.38 ± 0.01 ^e^	2.18 ± 0.01 ^a^
C*	19.44 ± 0.03 ^e^	23.54 ± 0.01 ^i^	15.78 ± 0.01 ^c^	23.00 ± 0.01 ^h^	34.20 ± 0.03 ^j^	12.21 ± 0.01 ^b^	22.01 ± 0.01 ^g^	18.60 ± 0.01 ^d^	19.48 ± 0.01 ^f^	8.27 ± 0.01 ^a^

Note: Different lowercase letters among the same group indicate significant differences (*p* < 0.05). Each value is expressed as mean ± standard deviation (*n* = 3).

## Data Availability

Data supporting the findings of this study are available within the manuscript/Supplementary Material. Should any raw data files be needed in another format they are available from the corresponding author upon request.

## Supplementary Materials

The following supporting information can be downloaded at: https://www.mdpi.com/article/10.3390/foods14244182/s1, Table S1: Sample product information.
