# Supplementary material for: Multivariate Analysis of Key Taste Compounds in Soy Sauce and Model Construction for Its Saltiness Intensity
Source: Foods. 2025 Dec 5;14(24):4182. doi: 10.3390/foods14244182 (PMC12731317; doi:10.3390/foods14244182)
Supplement: Supplementary file 1 [file foods-14-04182-s001.zip › foods-3995258-supplementary.pdf]

Table S1. Sample product information

Soy sauce	Fermentation substrate	Add MSG	Fermentation type	Ammonium nitrogen (g/100mL)	Quality grade	Na content (mg/15mL)
Z10-1	wheat, non-genetically modified defatted soybean	yes	high-salt diluted-state	≥ 1.20	special grade	885
Z10-2	wheat, wheat flour, non-genetically modified soybean	yes	high-salt diluted-state	≥ 0.50	third grade	1080
Z10-3	wheat, rice, non-genetically modified soybean	yes	high-salt diluted-state	≥ 1.00	first grade	1149
Z10-4	wheat, non-genetically modified soybean	yes	high-salt diluted-state	≥ 0.50	third grade	1150
Z10-5	wheat, wheat flour, non-genetically modified defatted soybean	yes	high-salt diluted-state	≥ 0.80	first grade	806
Z10-6	wheat, non-genetically modified soybean	yes	high-salt diluted-state	≥ 1.20	special grade	1083
Z10-7	wheat, wheat flour, non-genetically modified soybean	yes	high-salt diluted-state	≥ 0.40	third grade	1079
Z10-8	wheat, non-genetically modified soybean	no	high-salt diluted-state	≥ 1.20	special grade	938
Z10-9	wheat, non-genetically modified defatted soybean	yes	high-salt diluted-state	≥ 1.20	special grade	1070
Z10-10	wheat, non-genetically modified soybean	yes	high-salt diluted-state	≥ 1.20	special grade	1060
